# Prediction of Biomass and N Fixation of Legume–Grass Mixtures Using Sensor Fusion

**DOI:** 10.3389/fpls.2020.603921

**Published:** 2021-01-21

**Authors:** Esther Grüner, Thomas Astor, Michael Wachendorf

**Affiliations:** Grassland Science and Renewable Plant Resources, Organic Agricultural Sciences, Universität Kassel, Witzenhausen, Germany

**Keywords:** multispectral, point clouds, grassland, remote sensing, texture

## Abstract

European farmers and especially organic farmers rely on legume–grass mixtures in their crop rotation as an organic nitrogen (N) source, as legumes can fix atmospheric N, which is the most important element for plant growth. Furthermore, legume–grass serves as valuable fodder for livestock and biogas plants. Therefore, information about aboveground biomass and N fixation (NFix) is crucial for efficient farm management decisions on the field level. Remote sensing, as a non-destructive and fast technique, provides different methods to quantify plant trait parameters. In our study, high-density point clouds, derived from terrestrial laser scanning (TLS), in combination with unmanned aerial vehicle-based multispectral (MS) data, were collected to receive information about three plant trait parameters (fresh and dry matter, nitrogen fixation) in two legume–grass mixtures. Several crop surface height metrics based on TLS and vegetation indices based on the four MS bands (green, red, red edge, and near-infrared) were calculated. Furthermore, eight texture features based on mean crop surface height and the four MS bands were generated to measure horizontal spatial heterogeneity. The aim of this multi-temporal study over two vegetation periods was to create estimation models based on biomass and N fixation for two legume–grass mixtures by sensor fusion, a combination of both sensors. To represent conditions in practical farming, e.g., the varying proportion of legumes, the experiment included pure stands of legume and grass of the mixtures. Sensor fusion of TLS and MS data was found to provide better estimates of biomass and N_*Fix*_ than separate data analysis. The study shows the important role of texture based on MS and point cloud data, which contributed greatly to the estimation model generation. The applied approach offers an interesting method for improvements in precision agriculture.

## Introduction

Legume–grass mixtures, sown as temporary grassland and cultivated for 1–3 years, are substantial crop rotation elements, especially for organically managed farms in the European temperate climate. These crops are valuable forage for livestock and substrate for biogas plants. Furthermore, farmers utilize the ability of legumes to fix nitrogen (N), which is the main essential element for plant growth and health, to increase soil fertility and to reduce the amount of external fertilizer for the following cash crop (Fustec et al., 2010; Rasmussen et al., 2012). Total aboveground biomass and the amount of fixed N (N_*Fix*_), which contributes greatly to the N cycle on field and farm level, are important input variables for sustainable management decisions (Kayser et al., 2010). Traditional methods for grassland monitoring based on destructive biomass sampling, manual plant height measurement, and laboratory work are time and cost-intensive. Therefore, developments of non-destructive measurement techniques from the field of remote sensing provided interesting approaches and improvements for field data acquisition (Wachendorf et al., 2018).

Remote sensing was successfully used to estimate different biophysical and chemical plant traits in grasslands. As plant height correlates with biomass, canopy surface height (CSH) of grassland can be conducted by spatial information based on three-dimensional (3D) point clouds with an ultrasonic sensor (Fricke et al., 2011), Light Detection and Ranging (LiDAR) (Anderson et al., 2018; Xu et al., 2020) or Structure from Motion (SfM) based on RGB (red, green, blue) images (Wijesingha et al., 2018; Grüner et al., 2019). In a previous study of Grüner et al. (2019), SfM based on RGB images captured by an unmanned aerial vehicle (UAV) with a horizontal spatial resolution of ∼2 cm in two legume–grass mixtures was used for biomass estimation. The authors pointed out that a higher resolution and the inclusion of plant density information could increase model accuracy. Depending on the scanning angle and the range of the laser impulse, LiDAR can be used to generate a deeper point cloud penetration and higher spatial resolution and, therefore, can also cover single grass tillers (Cooper et al., 2017; Madec et al., 2017). However, the sole application of monochrome LiDAR systems generates only spatial characteristics of vegetation traits like mean, maximum, and median height metrics. Because of this, deriving information on biochemical vegetation characteristics is limited.

These restrictions can be compensated by spectral sensors, which quantify multi- and hyperspectral reflectance information and can be used to calculate vegetation indices (VIs) (Reddersen et al., 2014; Moeckel et al., 2017). A spectral sensor mounted on a low-cost UAV serves as an interesting and simple tool for grassland monitoring. VIs were already successfully used to estimate grassland biomass, N content, N_*Fix*_ (Cho et al., 2007; Gao et al., 2019; Grüner et al., 2020) and are highly correlated to leaf area index (LAI) (Mutanga and Skidmore, 2004; He et al., 2006; Darvishzadeh et al., 2008). However, the sole application of VIs for biomass estimation is affected by soil background color at low biomass levels (Huete et al., 1985) and saturates at high biomass and LAI levels (> 2–3) (Carlson and Ripley, 1997; Mutanga and Skidmore, 2004), as it captures reflectance only of the top surface of the canopy.

Consequently, sensor fusion of spatial and spectral information may overcome the limitations mentioned earlier and gained considerable interest as a new approach to assess forage yield and quality (Karunaratne et al., 2020). Most sensor fusion studies in grasslands utilizing CSH metrics were based on ultrasonic sensors (Fricke and Wachendorf, 2013; Moeckel et al., 2017; Gebremedhin et al., 2019) or UAV-based RGB SfM approaches (Geipel et al., 2014; Possoch et al., 2016; Lussem et al., 2019; Karunaratne et al., 2020). Although LiDAR provides high 3D point cloud resolution, the combination with a spectral sensor was only done by Schaefer and Lamb (2016) in a *Festuca arundinacea*-dominated grassland and never done for plant traits such as N fixation. The results of Wang et al. (2017) showed improved biomass estimation accuracy by LiDAR-based height metrics and VIs in maize, compared with models solely based on one sensor system. Similar results were found by Tilly et al. (2015) in a barley experiment using combined terrestrial laser scanning (TLS) and hyperspectral data. Therefore, the utilization of LiDAR, in combination with VIs, could further enhance the prediction accuracy of the forage parameter.

Grassland, as well as legume–grass mixtures, can be botanically, structurally, and phenologically very diverse (Cho et al., 2007; Schellberg et al., 2008; Biewer et al., 2009), as they consist of a composition of different species, compared with other crops, which are usually cultivated in monoculture. CSH metrics and VIs ignore this horizontal heterogeneity within vegetation. Texture features, derived from high-resolution images of vegetation, proved to serve additional structural information and correlate with heterogeneity (Gallardo-Cruz et al., 2012). The analysis of texture describes the spatial and statistical relationship of pixels (gray level values) and their variation in a defined area of interest in an image (Haralick et al., 1973; Wood et al., 2012). Texture features based on spectral data are sensitive to the phenological growth stage of the plant (Culbert et al., 2009) and increase data information of crop canopy without additional sensors. The inclusion of texture features for biomass and LAI estimation was mainly done in forests (Lu, 2005; Wijaya et al., 2010; Morin et al., 2019) and to a lower degree for crops such as rice (Li et al., 2019; Zheng et al., 2019, 2020) and wheat (Yue et al., 2017). In grasslands, the study of Grüner et al. (2020) investigated the influence of texture features based on multispectral (MS) data on model accuracy for biomass and N_*Fix*_ prediction in two legume–grass mixtures. The study clearly showed promising results for fresh (FM) and dry matter (DM) estimation, whereas, for N_*Fix*_, the results were not fully clear.

The present study aimed to develop a multi-temporal estimation model for aboveground biomass and N_*Fix*_ of two legume–grass mixtures. Estimation models were created using CSH metrics generated from TLS data and UAV-based MS data. Furthermore, texture features were extracted from MS and CSH data, which was never done before for grassland. As the study has a high number of predictors in combination with high multi-collinearity, a common machine learning algorithm, Random Forest (RF) (Breiman, 2001), in combination with a previous variable selection, was used for model generation (Belgiu and Drãguţ, 2016). Thus, the specific objectives of this study are: (1) The development of biomass (FM and DM) and N_*Fix*_ estimation models for clover– (CG) and lucerne–grass (LG) mixtures (0–100% legumes) based on two complete growing periods. (2) Comparing the exclusive model generation based on CSH from TLS and based on MS information from UAV-based MS imagery with the prediction model based on the fusion of both sensors. (3) Identifying the most important parameter for the prediction of the grass–legume mixtures and evaluate the contribution of texture features.

## Materials and Methods

### Experimental Site

**FIGURE 1:**
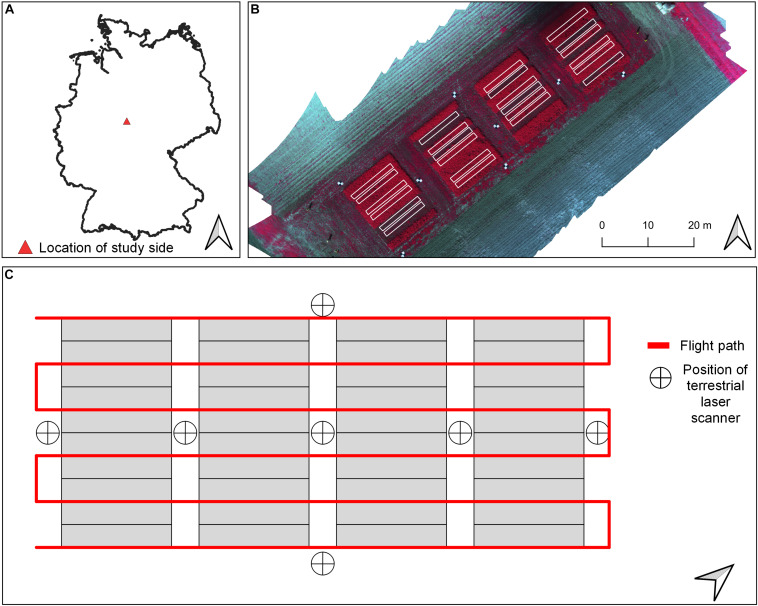
**(A)** Overview about location of study site. **(B)** Location of sampling plots used for analysis overlayed on a false-color (band combination: NIR, red, green) orthomosaic from May 2019. **(C)** Schematic view of flight plan used for spectral data collection (red line) and positions of terrestrial laser scans within the experimental layout.

The field study was carried out on a legume–grass experiment in two consecutive growing seasons, 2018 and 2019, which is located on the research farm of the Universität Kassel in Neu-Eichenberg (51 23’N, 9 54’E, 227 m asl.) in Hesse, Germany (Figure 1A). The mean annual precipitation and daily temperature of the site are 661 mm and 8°C, respectively, which was not reached for the study years, especially for 2018 (Table 1). The research farm is managed organically, and therefore, no fertilizer and chemicals were applied.

**TABLE 1:** Total rainfall, number of samples, and UAV flight information for both research years.

**Year**	**Annual rainfall (mm)**	**Harvest date**	**Harvest**	**Number of samples (*n*)**	**Flight mode**	**Flight altitude**
2018	380	17.05.18	First harvest (H1)	72	Manually	50 m
		20.06.18	Second harvest (H2)		Manually	50 m
		03.08.18	Third harvest (H3)		Manually	20 m
2019	641	23.05.19	First harvest (H1)	68	Autopilot	20 m
		04.07.19	Second harvest (H2)		Autopilot	20 m
		22.08.19	Third harvest (H3)		Manually	20 m

The study design was adapted from Grüner et al. (2020) and was continued for the growing season of 2019. Field plots (*n* = 24, size: 1.5 × 12 m^2^) were sown in autumn 2017 and cultivated for the following two study years with six different treatments in four replicates, which were mowed three times a year in accordance with the common agricultural practice within the region (Table 1 and Figure 1B). Due to unfavorable growing conditions at the third sampling date in 2019, four plots were excluded from further analysis. In total, 140 plots for the FM and DM modeling and 94 plots for N_*Fix*_ were included in statistical analysis.

The six treatments were composed of two legume–grass mixtures, CG and LG, and one pure stand of legumes (L_*CG*_ and L_*LG*_) and grass (G_*CG*_ and G_*LG*_) for each mixture with a seeding density of 35 kg ha^–1^ (Supplementary Table 1). CG included 60% *Lolium multiflorum*, 30% *Trifolium pratense*, 5% *Trifolium hybridum* L., and 5% *Trifolium repens* L., whereas LG consisted of 40% *Medicago sativa*, 20% *Festuca pratensis* Huds., 15% *Lolium perenne* L., 10% *L. multiflorum*, 10% *T. pratense*, and 5% *Phleum pratense* L.

### Data Acquisition

**FIGURE 2:**
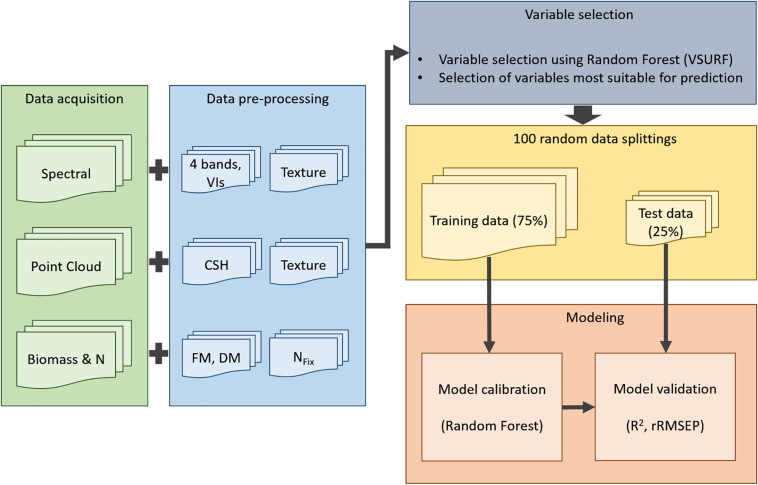
Workflow for model processing: Acquisition (green) of spectral information (green, red, red edge, NIR), of point cloud data from terrestrial laser scanning data and of reference data for biomass and N_*Fix*_; data preprocessing (blue): eight texture features of each spectral band (four bands), 13 vegetation indices (VI), 15 crop surface height (CSH) parameters, eight texture features of mean CSH, fresh (FM) and dry matter (DM) yield, and N_*Fix*_ calculation; variable selection (gray); 100 random data splittings (yellow); modeling (red).

TLS and UAV flight missions were done 1 day before every harvest. A Leica real-time kinematic (RTK) global navigation satellite system (GNSS) receiver with a measuring accuracy of 2 cm was used to measure the coordinates of the plot corners of every plot (Figure 1C). An overview of the workflow for data acquisition and processing is given in Figure 2.

### Terrestrial Laser Scanning Data Collection

A Leica ScanStation P30 (Leica Geosystem, Switzerland) was used for the point cloud data collection. The laser transmits and captures 1 million pulses per second at a wavelength of 1,550 nm with a resolution of 3.2 mm at a 10 m distance. For each harvest date, seven scans were taken, distributed in the experimental field between the blocks and at the four borders of the field to cover the plots from each side (Figure 1C). The laser was mounted on a tripod at the height of approximately 1.70 m. Three reflective control points were used at every scan for the later point cloud alignment of the digital surface model (DSM). The Leica RTK GNSS receiver measured the coordinates of the control points. One additional TLS data set was generated after the first harvest of the first study year for deriving the digital elevation model (DEM).

### Multispectral Data Collection

Spectral images were taken in the morning (8:00–12:00 a.m.) with an MS sensor (Parrot Sequoia, MicaSense Inc, Seattle, United States) mounted on a UAV (2018: DJI Phantom 3, Advanced; 2019: DJI Phantom 4 Professional, Shenzhen, China). The sensor captures 1.2-megapixel images in four bands [green: 530–570 nm; red: 640–680 nm; red edge: 730–740 nm; near-infrared (NIR): 770–810 nm]. For automatic radiometric calibration of every image, an upwelling sunshine sensor on the top of the UAV measures the at-the-sensor irradiance. The UAV was flown manually, except for the first two cuts in 2019, where the autopilot (Pix4Dcapture, Lausanne, Switzerland) was used (Table 1). Seven ground control points (GCP) were evenly distributed in the pathways between and around the plots. The Leica RTK GNSS receiver measured the coordinates of the GCPs.

### Biomass Sampling and N Fixation Determination

In 2018, the first 1.5 m of every plot was used for destructive measurements between the main harvests (Grüner et al., 2020). Therefore, this area was excluded for biomass sampling and data processing in every plot in both years. At each harvest date, two destructive samples of 0.25 m^2^ were taken from every plot, which were weighed for FM determination and afterward dried at 100°C for 48 h to constant weight to determine the DM content. For further analysis, weights were extrapolated to tonnes per hectare. Additional subsamples of every plot were taken for N concentration analysis, which was quantified by an elemental microanalyzer (Elementar vario MAX CHN, Langenselbold, Germany) and multiplicated with DM yield. To determine N_*Fix*_ of the legumes and the mixtures, the difference method, according to Stülpnagel (1982), was used (Eq. 1):

(1) N=F⁢i⁢xN-LNR

where N_*L*_ is the amount of N of legume in the pure stand and in the mixture as the N fixing crop, whereas N_*R*_ represents the amount of N of the pure stand of grasses as the non-fixing reference crop. Four samples from 2019 were not generated due to problems in the laboratory (Table 1).

## Data Preprocessing

### Crop Surface Height Parameter

The point cloud processing software Leica Cyclone 3D (Leica Geosystem, Glattbrugg, Switzerland) was used for merging and geo-referencing the point clouds of the TLS data sets using the GCPs. After exporting the point clouds, R version 3.5.1 (R Core Team, Vienna, Austria) was used for further computation. To convert the 3D point cloud to 2D height information for the DSMs and DEM of every plot, a raster with a 5 cm cell size was overlaid, and the height values of the points (*z*-values) within each cell were extracted. Due to geo-referencing, the DEM fitted accordingly to the DSMs and was subtracted from each other to calculate the CSH for every plot and harvest date (Eq. 2):

(2) C⁢S⁢H=D⁢S⁢M-D⁢E⁢M

In addition to the arithmetic mean CSH value of every plot, the minimum (MIN), maximum (MAX), median, variance, standard deviation, range, mode, skewness, kurtosis, canopy height relief (based on Silva et al., 2017), and the percentiles of 25, 75, 90, and 95% were computed and averaged for each plot (18 m^2^).

### Multispectral Bands and Vegetation Indices

For photogrammetric processing, Agisoft PhotoScan Professional (Agisoft LLC, St. Petersburg, Russia) was used for MS orthomosaic generation. After alignment of the overlapping images of each data set, a sparse point cloud was created with the accuracy setting “high” and a key point and tie point limit of 40,000 and 1,000, respectively. The accuracy of the sparse point cloud was enhanced by including GPS coordinates of the GCPs and automatic camera calibration. To generate a dense point cloud, parameter settings were set to “high” with a “mild” depth filtering. As flight height varied (20 and 50 m), in the last step, the MS orthomosaics were exported as a tagged image file format with a 4.5 cm ground resolution for unified conditions. To extract spectral information of every band for every plot, zonal statistics in Quantum Geographical Information System (QGIS 3.4.9, QGIS Development Team, Raleigh, NC, United States) was used by creating polygon masks for each plot. Additional to the four spectral bands, 13 VIs were used in this study (Supplementary Table 2). VIs were calculated with the original spectral mean value of every plot.

### Texture Features

Haralick et al. (1973) proposed 14 texture features for the gray level co-occurrence matrix of the image texture. Based on the study of Grüner et al. (2020), eight of these gray level co-occurrence texture features were used (Supplementary Table 3). In QGIS, these eight features were provided by the Orfeo Toolbox library (OTB, open-source; Grizonnet et al., 2017; Morin et al., 2019), i.e., energy, entropy, correlation, inverse difference moment, inertia, cluster shade, cluster prominence, and Haralick correlation (Supplementary Table 3). Texture feature extraction was done for the mean CSH and the four spectral bands (green, red, red edge, and NIR), keeping settings on default, “simple” texture set, and a radiometric resolution of 16 bits. In the final step, the average of every texture feature was calculated for each plot.

### Statistical Modeling and Variable Selection

Biomass (FM and DM) and N_*Fix*_ were predicted based on the height parameter (CSH including texture features) and MS reflectance information (MS, including VIs and texture features). The third model was based on a data fusion of the CSH and spectral information (Fusion). Before each modeling step, a variable selection based on the three-step procedure as suggested by Genuer et al. (2010) was conducted to identify the most important variables with the strongest relationship to the dependent variables (i.e., FM, DM, and N_*Fix*_). In the first step (“thresholding step”), irrelevant variables were removed by calculating the importance of each variable in 50 random forest model runs (no model optimization is applied). In the second step (“interpretation step”), the most informative variables are selected based on the out-of-bag (OOB) error of 25 random forest model runs. For the third step (“prediction step”), the ranked variables from the second step were added to the final model only if the OOB error decreased significantly more than the average variation obtained by adding noisy variables. The calculations were done using the *VSURF* package (Genuer et al., 2015) in the software environment R. RF was used from the R packages *caret* (Kuhn, 2008) and *randomForest* (Liaw and Wiener, 2002). For the optimization of the RF models using the selected variables, the data sets were divided into two subsets, where a calibration data set (75%) was used for model calibration, and the remaining data set (25%) was used as validation data set. To reduce the effect of autocorrelations between samples (e.g., samples from the same treatment are more similar than samples from different treatments) and to reduce the risk of overfitting, the data splitting was done randomly 100 times. For the splitting, it was ensured that the validation data set always contained samples of each year, harvest date, and treatment (Roberts et al., 2017). During the validation procedure, each sampling point was on average (i.e., based on median) 25 times in the validation data set. For model calibration, a cross-validation for hyper-parameter tuning of *mtry* was done, which represents the number of randomly chosen variables. *Mtry* was set by dividing the number of samples (*n*) by 3 as recommended by Probst et al. (2018), where *n* was 140 for FM and DM (Table 1) and 94 for N_*Fix*_ (excluding pure grass-plots). For model validation, the model performance between observed and predicted FM, DM, and N_*Fix*_ was calculated using the coefficient of determination of the validation (*R*^2^_*v*__*al*_) (Eq. 3) and the relative root mean squared error of prediction (rRMSEP) (Eq. 4).

(3) $$R_{v\,a\,l}^{2}=\left[1-\frac{\sum_{i=1}^{n}{\left(y_{i}-{\hat{y}}_{i}\right)}^{2}}{\sum_{i=1}^{n}{\left(y_{i}-{\bar{y}}_{i}\right)}^{2}}\right]$$

(4) $$r\,R\,M\,S\,E\,P=\frac{\sqrt{\frac{\sum_{i=1}^{n}{\left(y_{i}-{\hat{y}}_{i}\right)}^{2}}{n}}}{m\,a\,x\,\left(y_{i}\right)-m\,i\,n\,\left(y_{i}\right)}$$

where *y* is the observed and $\hat{y}$ the predicted value, $\bar{y}$ the average predicted value, and *n* the sample size. To determine the variable importance, the variables in all 100 models were sorted by the median importance value. The importance value is the mean of squared residuals (mean squared error), which is the difference between calculated on OOB data for every decision tree and permuted for each variable (Liaw and Wiener, 2002; Kuhn, 2008). To examine the effect of sampling year on the prediction quality, the normalized deviation of the predicted from the observed values (norm.dev) was calculated (Eq. 5). The resulting values are scaled from −1 to 1, indicating underestimation and overestimation, respectively. Subsequently, by using a Kruskal–Wallis test and a pairwise comparison with the Dunn test, the effect of year for each legume–grass mixture (CG and LG) on the normalized deviation (i.e., deviation of predicted from observed values) was calculated. Using this method, a systematic effect of sampling year on the prediction quality could be evaluated.

(5) $$norm.dev.=\frac{{\hat{y}}_{i}-y_{i}}{{\hat{y}}_{i}+y_{i}}$$

where *y* is the observed and $\hat{y}$ the predicted value.

## Results

### Ground Truth Data

**FIGURE 3:**
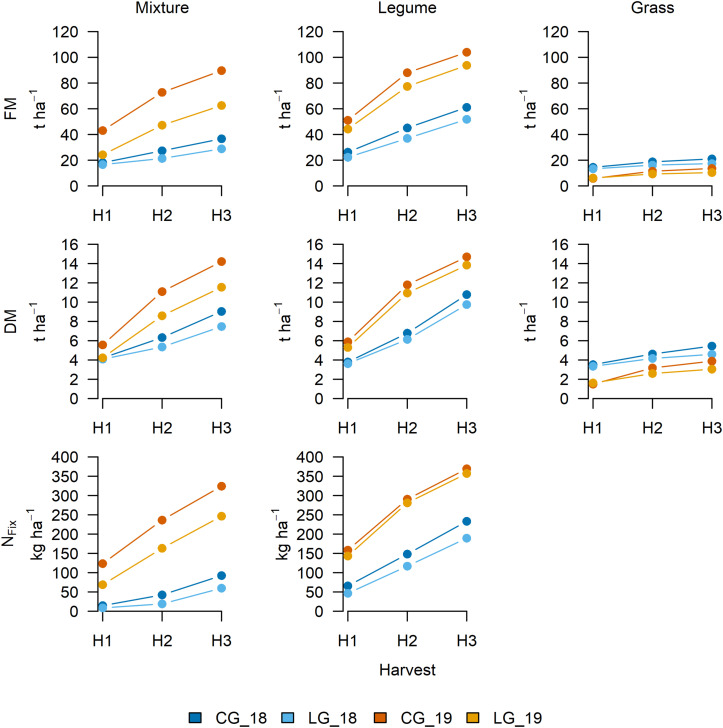
Cumulative fresh matter (FM) and dry matter (DM) yield and nitrogen fixation (N_*Fix*_) after first (H1), second (H2: H1 + H2), and third (H3: H1 + H2 + H3) harvest in each of the two study years (2018 and 2019) for clover– (CG) and lucerne–grass (LG) mixtures (left) and pure stands of legumes (middle) and grass (right) as included in mixtures.

In both study years, all values for FM, DM, and N_*Fix*_ of CG (mixture, legume, and grass) exceeded those for LG (Figure 3). Due to severe drought in 2018, biomass and N_*Fix*_ were higher in 2019 for both CG and LG in the mixture and its legume, whereas the grass showed the opposite. As no fertilizer was applied, the grass suffered from nutrient deficiencies, especially in the second growing period. The average annual FM yield after the third harvest (H3) ranged between 10.36 for G_*LG*__19 and 103.94 t ha^–1^ for L_*CG*__19 and DM yield between 3.05 for G_*LG*__19 and 14.70 t ha^–1^ for L_*CG*__19. The average N_*Fix*_ (H3) varied between 59.73 for LG_18 and 369.24 kg ha^–1^ for L_*CG*__18.

### Biomass and N_*Fix*_ Prediction

The selection of the most important variables for each data set (i.e., CSH, MS, and fusion) resulted in a reduced number of variables for each dependent variable (i.e., FM, DM, and N_*Fix*_). Although for FM, the number of variables was nine for CSH and MS and eight for the fusion data set, for DM, the number of variables ranged from 10 for CSH to 14 for the fusion data set. For N_*Fix*_, the lowest number of variables was found for CSH (*n* = 6) and the largest for fusion (*n* = 11).

**FIGURE 4:**
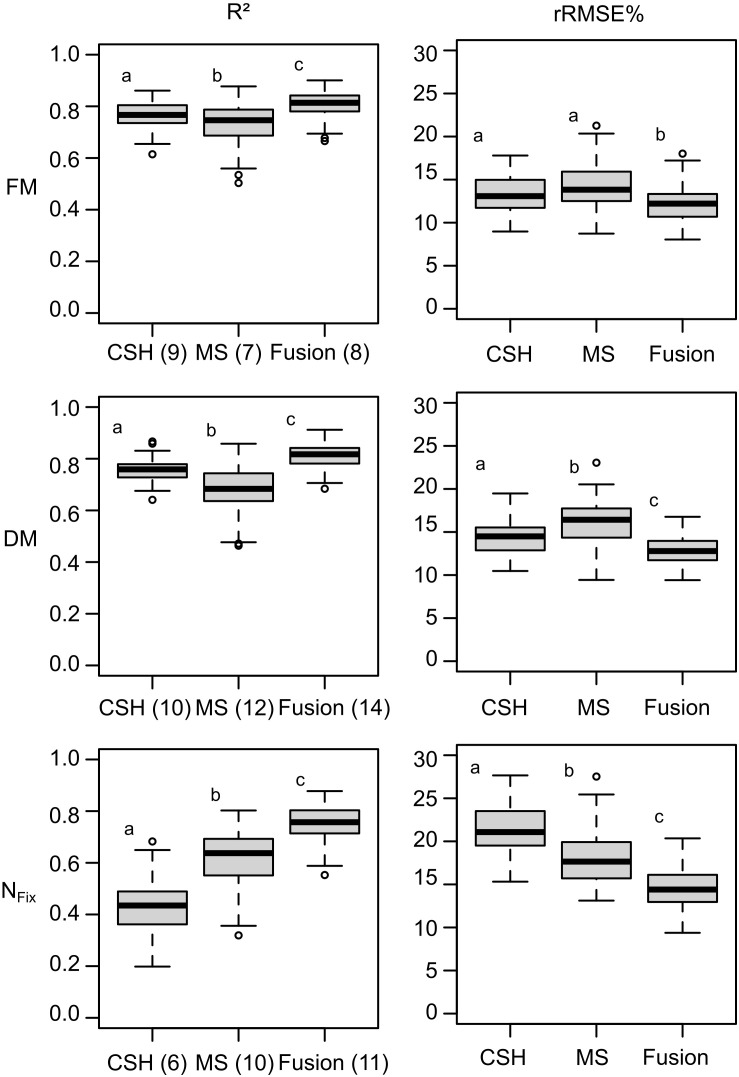
Boxplots for model accuracy based on 100 randomly divided calibration (75%) and validation (25%) for whole data set, including clover– and lucerne–grass as mixtures and pure stands of legume and grass of mixtures. Model generation was done with most important variables selected during variable selection procedure from crop surface height (CSH) information, including texture features, from multispectral (MS) information, including texture and vegetation indices. Numbers in brackets indicate number of selected variables used for model optimization. Boxes show 25 and 75% percentiles; solid line indicates median; whiskers represent 5 and 95% percentiles; circles show extreme values.

The prediction accuracy of the models based on 100 random data splitting for calibration and validation is shown in Figure 4. For FM, CSH prediction models performed better than MS with a median rRMSEP of 13.08 and 13.82%, respectively. Sensor fusion showed the best model accuracy with an *R*^2^ of 0.81 and an rRMSEP of 12.20%. Similar to FM, CSH showed a lower rRMSEP of 14.49% for DM compared with MS with 16.42%. The best model performance with an *R*^2^ of 0.82 and an rRMSEP of 12.79% was found for the sensor fusion data set. Again, the model improvement was statistically significant based on a Kruskal–Wallis test with a pairwise comparison using the Dunn test. For N_*Fix*_, MS showed the better model accuracy with an rRMSEP of 17.64% compared with CSH with 21.07%, although the best model was achieved again by sensor fusion with an *R*^2^ of 0.76 and an rRMSEP of 14.40%.

**FIGURE 5:**
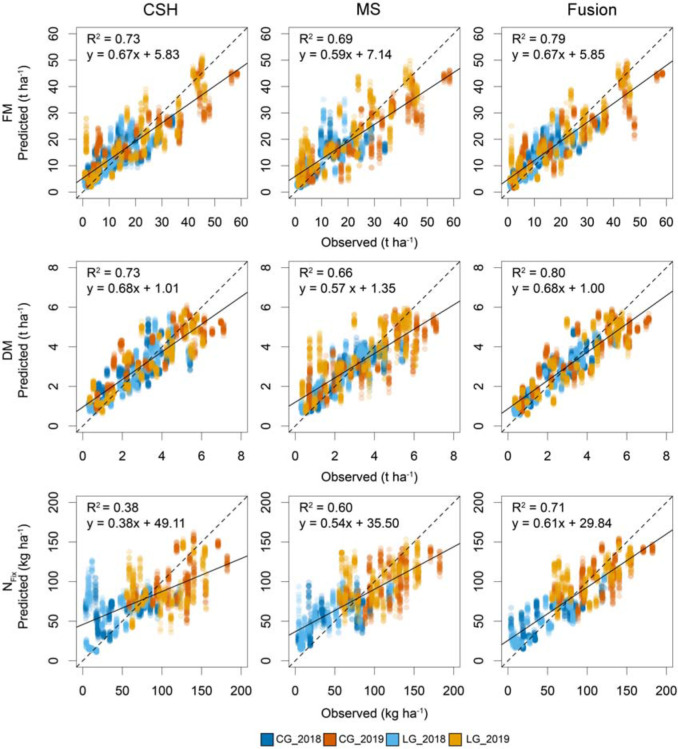
Scatterplot based on 100 randomly divided calibration (75%) and validation (25%) for observed and predicted fresh (FM) and dry matter (DM) yield and nitrogen fixation (N_*Fix*_) for whole data set at each of two study years (2018 and 2019) including clover– (CG) and lucerne–grass (LG) as mixtures, including corresponding pure stands of legume and grass of mixtures. Dotted line indicates 1:1 diagonal, whereas solid line shows regression line.

The plot of fit (Figure 5) of the 100 model runs for FM and DM showed no clear pattern for CSH and MS. Only a slight overestimation at low and underestimation at high yields was visible. The fusion of CSH and MS reduced this. For N_*Fix*_, the over- and underestimation at low and higher yields, respectively, were stronger, especially for CSH. This was also reduced by sensor fusion.

**FIGURE 6:**
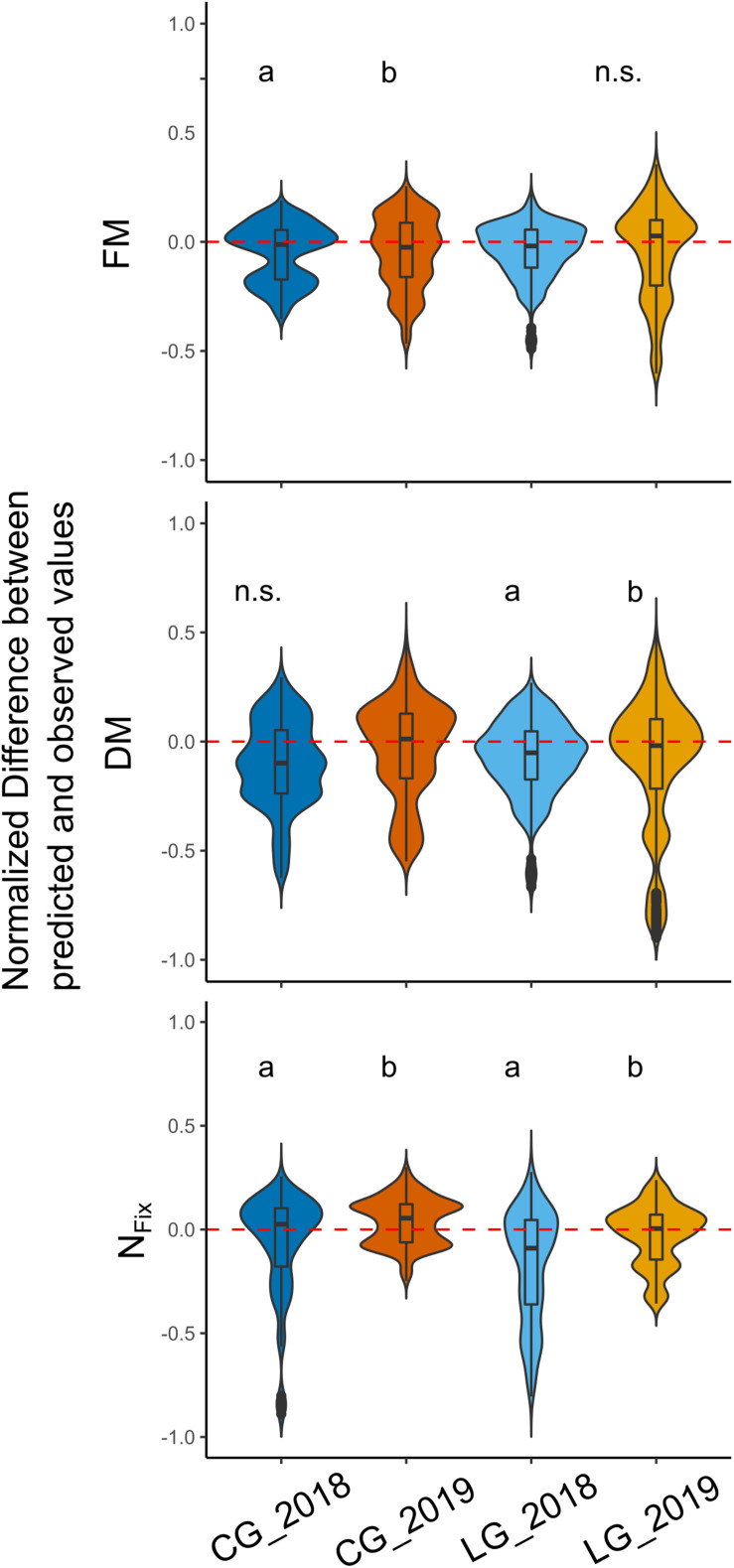
Distribution of normalized deviations between predicted and observed values for each legume–grass mixture (blue: clover–grass, orange: lucerne–grass) and sampling year (dark: 2018, bright: 2019). Width of boxes indicates frequency distribution of values. Dotted line characterizes level of zero deviation (i.e., 100% correct prediction). Different letters indicate significant differences between years (based on Kruskal–Wallis and Dunn test). n.s. indicates that no significant differences in respect to sampling year were found.

Additionally, the effect of sampling year on the deviation of the predicted from the observed values for FM, DM, and N_*Fix*_ was examined (Figure 6). The results showed a significant difference between the FM prediction of CG in 2018 and 2019, with an overestimation in 2018 and a slight underestimation in 2019. For LG, no significant effects were found. For the DM predictions, no significant differences for CG were found, whereas, for LG, the differences were statistically significant (*p* < 0.01). For N_*Fix*_, the effect of year was statistically significant for both legume–grass mixtures (Figure 6).

**FIGURE 7:**
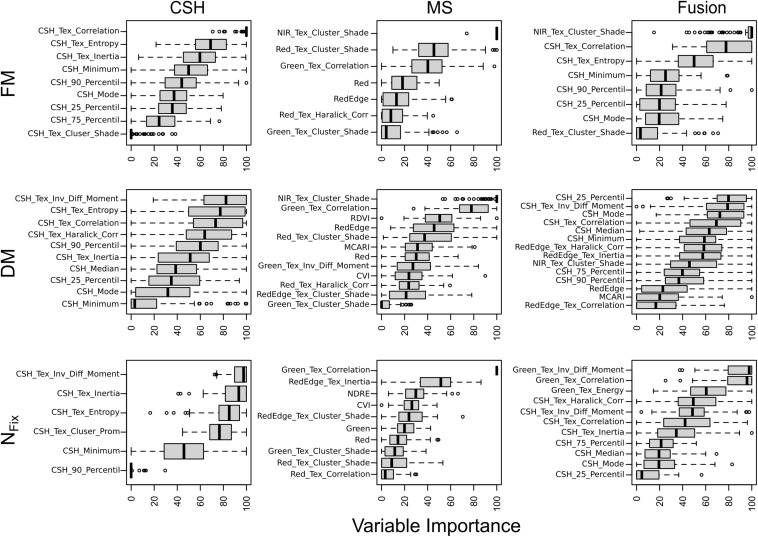
Importance of variables in prediction models based on 100 randomly divided calibration (75%) and validation (25%) for fresh (FM) and dry matter (DM) and nitrogen fixation (N_*Fix*_) for whole data set including clover– and lucerne–grass as mixtures and pure stands of legume and grass of mixtures. Model generation was done with crop surface height (CSH) information, including texture features, with multispectral (MS) information including texture and vegetation indices and with sensor fusion (Fusion) based on both CSH and MS. Boxes show 25 and 75% percentiles; solid line indicates median; whiskers represent 5 and 95% percentiles; circles show extreme values.

The importance of the RF model predictors (sorted by median) is separately shown for CSH and MS as well as for the sensor fusion in Figure 7. For FM, DM, and N_*Fix*_, the variable importance for CSH showed that height parameter (e.g., average height of all points from 90% percentile) and texture information (e.g., entropy) are relevant for the prediction. For MS, the texture information of the NIR band made the greatest contribution for both FM and DM. In contrast, for N_*Fix*_, the texture of the green band showed the highest importance (Figure 7). For FM and DM, the most important predictors for sensor fusion were CSH predictors, but also MS predictors, especially the NIR band including texture. For N_*Fix*_ sensor fusion, the texture features, especially from the green band, made the greatest contribution. Apart from the sensor fusion model for DM, VIs did not show any importance.

## Discussion

The aim of the current multi-temporal study was the development of estimation models for biomass and N_*Fix*_ of two legume–grass mixtures based on structural and spectral remote sensing information. RF, like other machine learning algorithms, needs a substantial amount of ground truth data, on the one side for calibration, but of similar importance, for validation of the model (Breiman, 2001). Multi-temporal studies, which cover a wider range of plant composition, yield, and vegetation periods, are essential for model development (Psomas et al., 2011; Ali et al., 2017). To our best knowledge, studies using machine learning based on UAV MS or TLS data for biomass prediction in grassland use only 1 year data, such as Capolupo et al. (2015); Viljanen et al. (2018), Anderson et al. (2018), Grüner et al. (2020), and Xu et al. (2020). Although Askari et al. (2019) generated UAV-based MS data for 2 years, each year included a different site. The present study, in contrast, consists of data based on two vegetation periods (i.e., three cuts each year) of the same experimental site. Furthermore, our study covers two legume–grass mixtures, typical for the European climate including a wide range of legume proportion (0–100%) of these mixtures, which makes our models transferable to practical farming.

Model generation was first done separately for each sensor system. For biomass estimation, CSH (14–15% rRMSEP) performed slightly better than MS (15–17% rRMSEP). Xu et al. (2020) used TLS data for aboveground biomass estimation in a heterogeneous permanent grassland and showed that TLS measurements are less affected by saturation than VIs as the laser infiltrates deeper into the vegetation. This might explain the advantage of TLS toward MS in this study. Grüner et al. (2020) gained an rRMSEP of 10–11% for sole MS data but containing subsamples between the harvests from 1 year, which may have created more robust but also less generalizable models.

Sensor fusion of CSH and MS significantly improved estimation model accuracy (12–13% rRMSEP). Our finding broadly supports the work of other studies in this area, linking crop height with MS information in grassland. Schaefer and Lamb (2016) used LiDAR for CSH and an optical reflectance sensor for measuring NDVI in a *F. arundinacea*-dominated grassland. The sensors were both mounted on a wheeled vehicle, 1.8 m above ground, where sensor fusion reduced RMSEP of 46 and 36%, respectively, in a linear regression model. Lussem et al. (2019) gained cross-validation results from multivariate linear regression of different VIs each combined with CSH from UAV RGB (90% percentile) with similar *R*^2^ between 0.57 and 0.75 for FM and *R*^2^ of 0.41 and 0.81 for DM, strongly depending on harvest date. The study of Viljanen et al. (2018) in a grassland experiment with different N fertilizing levels showed that the best model performance was given by a combination of VIs, RGB, and CSH features (rRMSE = 11–15%) for multilinear regression and RF. Näsi et al. (2018) could not confirm that biomass prediction by RF of grassland based on spectral and structural (both UAV RGB) parameters performed better than separate models. Nevertheless, rRMSE was on a very low level (2–6%) due to the low sample size (*n* = 8) with little variability, which limits its comparability and needs further investigations. Due to the severe drought in 2018 and missing fertilizer, mature grass was growing high with a very low amount of biomass, compared with mixtures and pure stands of legumes (Grüner et al., 2020). Fricke and Wachendorf (2013) showed that spectral information could compensate for the overestimation of CSH at low biomass levels, which might be a possible explanation of the benefit of sensor fusion in our study. For N_*Fix*_, MS (rRMSEP = 18%) performed better than CSH (rRMSEP = 21%). MS results are consistent with the measurements of the study of Grüner et al. (2020) (rRMSEP = 18%). N fixation is highly correlated to DM of legumes (Carlsson and Huss-Danell, 2003; Høgh-Jensen et al., 2004) and consequently also to crop height. Sensor fusion for N_*Fix*_ estimation further increased model accuracy (rRMSEP = 14%) in our study. The deviations between the predicted and the observed values confirm the expectations that a multi-temporal data set is needed for stable modeling results. Although a broad range of values for biomass and nitrogen fixation were collected in the present study, changing weather conditions or different agricultural practices might affect the predictions and need to be covered separately.

The results show that the importance of the variables differs between FM, DM, and N_*Fix*_ for the two sensors and their fusion. Apart from mean values for crop height and reflectance from the four spectral bands, our models contain several different parameters from both sensor systems. The most important variable of CSH and MS was based on texture features for FM, DM, and N_*Fix*_. Similar findings for MS were found by Grüner et al. (2020), which is the only study so far, including texture features based on spectral information for biomass and N_*Fix*_ estimation of legume–grass mixtures. Our results further support findings in other crops. Yue et al. (2019) used texture for wheat aboveground biomass estimation based on UAV RGB imaging with an *R*^2^ of 0.89 (RMSE = 0.82 t ha^–1^) by multiple stepwise regression. A recent study by Zheng et al. (2020) clearly showed an improvement of N content estimation for rice by combining VIs and texture features by UAV-based MS data.

To our best knowledge, our study is the first extracting texture features from TLS data in agricultural grasslands and, furthermore, combining them with spectral information. Texture detects other characteristics of plant structure than CSH and MS, especially differences in plant growth stages (Gao et al., 2019) and yield levels (Yue et al., 2019). Therefore, this supplementary information improves biomass and N_*Fix*_ estimation.

Both sensors, for TLS point clouds and UAV MS imaging, have their specific limitations, as they detect and measure different biophysical and chemical properties of vegetation and, furthermore, in our study from different altitude and view angle positions (nadir vs. oblique). TLS covers the area of interest in different distances within one scan due to the static measurement position, whereas UAV-based measurements are constant at equal flight altitude for the whole area. Therefore, UAV-based RGB imaging for crop height measurement by SfM in combination with MS data might have advanced handling; nevertheless, point density and accuracy are lower than for TLS (Wijesingha et al., 2018). As technical and computable improvement increases rapidly, a higher image resolution is expected in the next years for spectral sensors. Due to technical issues with the UAV software, except of two flights, all remaining flights were performed manually, which leads to uneven image overlapping. Further studies must overcome these uncertainties for unified flight missions and later analysis. Furthermore, image resolution plays a crucial role in texture feature extraction. In our study, MS resolution was 4.5 cm. Yue et al. (2019) showed in a winter-wheat experiment that image resolution between 5 and 15 cm showed an only low correlation between texture and aboveground biomass due to mixed pixels of soil and green vegetation. As legume–grass is rather heterogeneous compared with cereals, an image resolution enhancement could improve texture accuracy, which needs further research. In our study, the MS sensor covered specific wavelengths of green, red, red edge, and NIRS region. As the red edge region shifts to longer wavelengths for senescent material compared with green vegetation (Gao et al., 2019), a hyperspectral sensor can cover a much broader area of wavelengths. However, this approach needs more cost-intensive equipment and knowledge compared with MS sensors.

## Conclusion

Non-destructive quantification of plant traits in the grassland by remote sensing on the field-level enables the farmer to evaluate the *status quo* and to make prompt farm management decisions. The present study differs from previous studies in respect of (i) using CSH based on TLS in combination with MS data for sensor fusion, (ii) extracting and including texture features based on both TLS and MS information, and (iii) using multi-temporal data based on two vegetation periods of two legume–grass mixtures. The study showed that sensor fusion increased estimation model accuracy compared with separate sensor utilization and was a suitable method for estimating biomass and N fixation in two legume–grass mixtures. Sensor fusion provides a method to overcome the limits of each sensor and to improve prediction model accuracy. The variable importance analysis revealed that from a large number of available parameters, the texture was important input information. Furthermore, texture features can be easily implemented to the model, as no additional sensor is required. Selection of the most suitable texture feature for biomass and N_*Fix*_ estimation is important for model performance and can simplify model understanding. Nevertheless, feature selection of the optimal combination of height metrics and texture features still needs further research, as they performed differently for FM, DM, and N_*Fix*_.

Our approach is not yet feasible for practical farming, as TLS measurements are very time consuming and need advanced technical know-how. Nevertheless, with increasing technical and digital improvements in remote sensing, sensor fusion has great potential. In particular, further development of the UAV technique will allow sampling at field scale (with an area of several hectares), which is a prerequisite for any practical application of the proposed methods. Future research should focus on enhanced point cloud density and implementation of a UAV-based sensor system, which includes both CSH and MS information. Furthermore, a temporal resolution to provide a more holistic model and a deeper understanding of plant traits throughout the vegetation phase are necessary, especially for heterogeneous vegetation. The applied approach offers an interesting method for improvements in precision agriculture also for large areas.

## Data Availability Statement

The raw data supporting the conclusions of this article will be made available by the authors, on request without undue reservation.

## Author Contributions

EG conducted the data collection and processed the data. All authors contributed to the design, data analysis of the study, and writing and revision of the manuscript.

## Conflict of Interest

The authors declare that the research was conducted in the absence of any commercial or financial relationships that could be construed as a potential conflict of interest.
