# Supplementary material for: Prediction of Biomass and N Fixation of Legume–Grass Mixtures Using Sensor Fusion
Source: Front Plant Sci. 2021 Jan 21;11:603921. doi: 10.3389/fpls.2020.603921 (PMC7883874; doi:10.3389/fpls.2020.603921)
Supplement: Supplementary file 1 [file Data_Sheet_1.docx]

Supplementary Material

# Supplementary Tables

Table S1: List of treatments

| Treatment |  | Functional group | Species | Ratio (%) |
| --- | --- | --- | --- | --- |
| Clover-grass mixture | CG | Legumes (L) | *Trifolium pratense* | 30 |
|  |  |  | *Trifolium hybridum* | 5 |
|  |  |  | *Trifolium repens* | 5 |
|  |  | Grass (G) | *Lolium multiflorum* | 60 |
| Lucerne-grass mixture | LG | L | *Medicago sativa* | 40 |
|  |  |  | *Trifolium pratense* | 10 |
|  |  | G | *Festuca pratensis* | 20 |
|  |  |  | *Lolium perenne* | 15 |
|  |  |  | *Lolium multiflorum* | 10 |
|  |  |  | *Phleum pratense* | 5 |
| Pure clover legumes | L_CG_ | L from CG mixture | *Trifolium pratense* | 75 |
|  |  |  | *Trifolium hybridum* | 12.5 |
|  |  |  | *Trifolium repens* | 12.5 |
| Pure lucerne and clover legumes | L_LG_ | L from LG mixture | *Medicago sativa* | 80 |
|  |  |  | *Trifolium pratense* | 20 |
| Pure grass sward | G_CG_ | G from CG mixture | *Lolium multiflorum* | 100 |
| Pure grass sward | G_LG_ | G from LG mixture | *Festuca pratensis* | 40 |
|  |  |  | *Lolium perenne* | 30 |
|  |  |  | *Lolium multiflorum* | 20 |
|  |  |  | *Phleum pratense* | 10 |

Table S2: List of vegetation indices (VIs). Green (530-570 nm), Red (640-680 nm), RedEdge (730-740 nm), and NIR (770-810 nm) represent the wavebands used by the multispectral sensor to collection spectral information

| Name | Definition | Reference |
| --- | --- | --- |
| Simple Ratio | $SR= \frac{NIR}{Red}$ | (Jordan, 1969) |
| Modified Simple Ratio | $MSR= \frac{\frac{NIR}{Red}-1}{\sqrt{\frac{NIR}{Red}}+1}$ | (Chen, 1996) |
| Green Chlorophyll Index | $GCI= \left( \frac{NIR}{Green} \right)-1$ | (Gitelson et al., 2005) |
| Green Difference Vegetation Index | $GDVI= NIR-Green$ | (Tucker et al., 1979) |
| Difference Vegetation Index | $DVI=NIR-Red$ | (Tucker, 1979) |
| Normalized Difference Vegetation Index | $NDVI= \frac{NIR-Red}{NIR+Red}$ | (Rouse et al., 1974) |
| Renormalized Difference Vegetation Index | $RDVI= \frac{NIR-Red}{\sqrt{NIR+Red}}$ | (Roujean and Breon, 1995) |
| Green Difference Vegetation Index | $GNDVI= \frac{NIR-Green}{NIR+Green}$ | (Daughtry et al., 2000) |
| Normalized Difference Red Edge Index | $NDRE= \frac{NIR-RedEdge}{NIR+RedEdge}$ | (Fitzgerald et al., 2010) |
| Chlorophyll Vegetation Index | $CVI= \frac{NIR}{Green}*\frac{Red}{Green}$ | (Vincini et al., 2008) |
| Soil Adjusted Vegetation Index | $SAVI= \frac{NIR-Red}{NIR+Red+0.5}*\left( 1+0.5 \right)$ | (Huete, 1988) |
| Modified SAVI 2 | $MSAVI2= \frac{2*NIR+1-\sqrt{\left( 2*NIR+1 \right)^{2}-8*\left( NIR-Red \right)}}{2}$ | (Qi et al., 1994) |
| Modified Chlorophyll Absorption Index | $MCARI= \left[ \left( RedEdge-Red \right)-0.2*\left( RedEdge-Green \right) \right]*\frac{RedEdge}{Red}$ | (Daughtry et al., 2000) |

Table S3: Haralicks texture features

| Texture feature | Explanation |
| --- | --- |
| 1. Energy | Measures the local steadiness of the grey levels |
| 1. Entropy | Measures randomness or degree of disorder |
| 1. Correlation | Shows the linear dependency of grey level values in the GLCM |
| 1. Inverse Difference Moment | Measures the local homogeneity |
| 1. Inertia | Measures the local contrast or amount of variations |
| 1. Cluster Shade | Measures skewness of the GLCM |
| 1. Cluster Prominence | Measures the asymmetry of the GLCM |
| 1. Haralick Correlation | Shows the probability of two pixels with similar grey level |

**References**

Chen, J. M. (1996). Evaluation of vegetation indices and a modified simple ratio for

boreal applications. Canadian J. Remote Sens. 22, 229–242.

Daughtry, C. S. T., Walthall, C. L., Kim, M. S., Brown, de Colstoun, E., and

McMurtrey, J. E. (2000). Estimating corn leaf chlorophyll concentration from

leaf and canopy reflectance. Remote Sens. Environ. 74, 229–239. doi: 10.1016/

s0034-4257(00)00113-9

Fitzgerald, G., Rodriguez, D., and O’Leary, G. (2010). Measuring and predicting

canopy nitrogen nutrition in wheat using a spectral index—the canopy

chlorophyll content index (CCCI). Field Crops Res. 116, 318–324. doi: 10.1016/

j.fcr.2010.01.010

Gitelson, A. A., Vina, A., Ciganda, V., Rundquist, D. C., and Arkebauer, T. J. (2005).

Remote estimation of canopy chlorophyll content in crops. Geophys. Res. Lett.

32:L08403.

Huete, A. R. (1988). A soil-adjusted vegetation index (SAVI). Remote Sens. Environ.

25, 295–309. doi: 10.1016/0034-4257(88)90106-x

Jordan, C. F. (1969). Derivation of leaf-area index from quality of light on the forest

floor. Ecology 50, 663–666. doi: 10.2307/1936256

Qi, J., Chehbouni, A., Huete, A. R., Kerr, Y. H., and Sorooshian, S. (1994). A

modified soil adjusted vegetation index. Remote Sens. Environ. 48, 119–126.

doi: 10.1016/0034-4257(94)90134-1

Roujean, J.-L., and Breon, F.-M. (1995). Estimating PAR absorbed by vegetation

from bidirectional reflectance measurements. Remote Sens. Environ. 51, 375–

384. doi: 10.1016/0034-4257(94)00114-3

Rouse, J. W., Haase, R. H., Schell, A., and Deering, D. W. (1974). “Monitoring

vegetation systems in the great plains with ERTS,” in Proceedings of the Goddard

Space Flight Center 3d ERTS-1 Symp, (Washington, DC: NASA).

Tucker, C. J. (1979). Red and photographic infrared linear combinations for

monitoring vegetation. Remote Sens. Environ. 8, 127–150. doi: 10.1016/0034-

4257(79)90013-0

Vincini, M., Frazzi, E., and D’Alessio, P. (2008). A broad-band leaf chlorophyll

vegetation index at the canopy scale. Precision Agric. 9, 303–319. doi: 10.1007/

s11119-008-9075-z
